# Optimizing the screening process for TIRADS could reduce the number of unnecessary thyroid biopsies

**DOI:** 10.1530/EC-25-0097

**Published:** 2025-03-26

**Authors:** Ke Lu, Long Wang, Shuiqing Lai, Zhijiang Chen, Qibo Zhu, Shuzhen Cong, Kehong Gan, Xiaoyan Chen, Chunwang Huang, Jian Kuang

**Affiliations:** ^1^ Department of Endocrinology, Guangdong Provincial People’s Hospital (Guangdong Academy of Medical Sciences), Southern Medical University, Guangzhou, Guangdong, China; ^2^ Department of Ultrasound, Guangdong Provincial People’s Hospital (Guangdong Academy of Medical Sciences), Southern Medical University, Guangzhou, Guangdong, China; ^3^ Department of Endocrinology, First Affiliated Hospital of Guangzhou Medical University, Guangzhou, Guangdong, China

**Keywords:** thyroid ultrasound, TIRADS, fine-needle aspiration biopsy, missed malignancy rate, unnecessary biopsy rate

## Abstract

**Objective:**

Current Thyroid Imaging Reporting and Data Systems (TIRADS) exhibit considerable variability in size thresholds for fine-needle aspiration biopsy. This study harnesses the systematic variations among dissimilar TIRADS optimization strategies for biopsy selection.

**Methods:**

The analysis focused on the discrepancies observed among the four widely utilized TIRADS systems: ACR-TIRADS, Kwak-TIRADS, C-TIRADS and EU-TIRADS. Subsequently, several methods derived from the combination of two TIRADS were constructed via serial testing. Last but not least, diagnostic performance was assessed through unnecessary biopsy rate (UBR), missed malignancy rate and the frequency of clinically significant missed diagnoses.

**Results:**

A total of 699 nodules were included in the study. The accuracy for nodules consistently recommended for biopsy by the four TIRADS was merely 50.8%. Without elevating the risk of missed diagnoses, which could potentially influence prognosis as per the current literature, for eligible nodules recommended for biopsy by original TIRADS, incorporating another TIRADS in serial could further reduce the number of biopsies by 7.8–19.2%.

**Conclusions:**

Conspicuous disparities exist in biopsy guidelines among TIRADS systems, urging increased caution among healthcare providers, particularly when they are extensively applied in patient evaluations. As evidently demonstrated by our research findings, combining recommendations from two TIRADS systems could effectively and safely lessen UBRs. These findings also advocate for the integration of prognostic-impact assessment in developing novel biopsy optimization strategies.

## Introduction

Evaluating thyroid nodules is predominantly employed to distinguish between benign and malignant cases. On top of that, thyroid ultrasound has been regarded as the cornerstone of the current comprehensive management process (1). Suspicious thyroid nodules were usually evaluated by two-dimensional grayscale ultrasound to determine the risk category before the recommendation for fine-needle aspiration biopsy (FNAB), surgery or follow-up. This approach was principally reliant on the features of nodules, which comprise composition, orientation, margin, echogenicity and calcification. These features possess a high degree of discriminatory power. Nonetheless, relying solely on any individual feature alone makes it challenging to accurately determine the nature of a nodule. The Thyroid Imaging Reporting and Data Systems (TIRADS) system built on these features is a valuable tool under the circumstances. TIRADS is advantageous for dealing with multifarious problems such as variable interobserver reproducibility, lack of standardized reports, and intra- or inter-observer variability. The accuracy of distinguishing malignancy from benign cases could reach up to 80–90% (2, 3, 4).

To guide further treatment and to avoid unnecessary biopsy or surgery for benign nodules, another vital function of TIRADS is to recommend appropriate nodules for FNAB. Almost all currently published risk stratification systems had size thresholds for FNAB. Nevertheless, their criteria were diverse and controversial (1, 5, 6, 7, 8). Nodules measuring 1.5–2 cm displayed no correlation with augmented deaths and distant metastasis in comparison with 1.0–1.5 cm ones (9). Nguyen *et al.* demonstrated low local invasion and distant metastasis for smaller than 4 cm differentiated thyroid cancers but heightened all-cause mortality for all cancer categories larger than 2.5 cm (10). Nonetheless, it has also been noted that both papillary thyroid carcinoma (PTC) and follicular thyroid carcinoma (FTC) have a similar cumulative risk of metastasis for thyroid nodules larger than 2 cm (11). As a typical score-based system, ACR-TIRADS is manifested by high diagnostic specificity, which is further strengthened by high size thresholds for TR3 (≥2.5 cm) and TR4 (≥1.5 cm) categories (12). Essentially, establishing a size threshold is one approach to elevate the specificity of recommendations for undergoing FNAB. Is there any other way? Serial testing is an extensively utilized approach in diagnostic testing that improves specificity by combining several elements. We hypothesized that combining multiple TIRADS would be equally effective.

In this study, we probed deep into the discrepancies among the four TIRADS about the recommendations for FNAB and then explored combining two TIRADS to heighten the accuracy of TIRADS recommendations via serial testing.

## Materials and methods

### Patients and nodule selection

**Figure 1:**
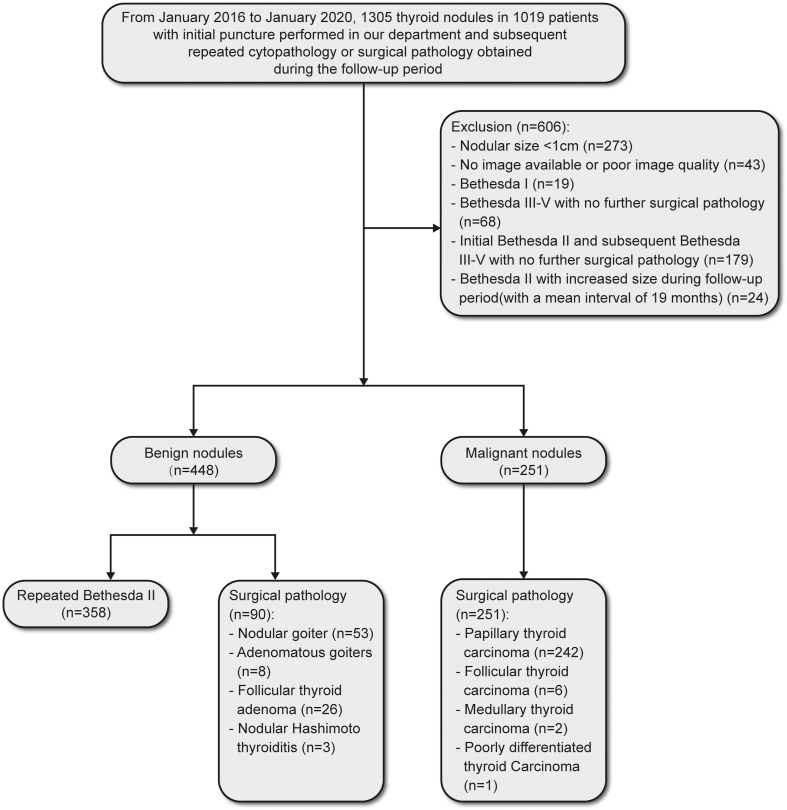
Study flow diagram.

This observational retrospective study enrolled patients with thyroid nodules who had definite diagnoses after an initial puncture at the Department of Endocrinology between January 2016 and January 2020. Benign diagnoses should be confirmed by repeat FNAB or surgical pathology. Malignant nodules should be diagnosed by surgical pathology. A total of 1,305 nodules in 1,019 patients were included. Two hundred seventy-three nodules were excluded due to a size smaller than 1 cm, and 179 nodules with only initial Bethesda II cytopathology and subsequent Bethesda III-V but without surgical pathology were also excluded. Twenty-four nodules with benign cytopathology were excluded due to a significant increase in size during the follow-up period (nodule growth is defined as the increase in two diameters >20% (and exceeding 0.3 cm) or volume >50%). Of the remaining 699 eligible nodules, 448 were diagnosed as benign, including 358 repeat Bethesda II and 90 benign surgical nodules. Surgical pathology confirmed 251 malignant nodules, including 242 PTC, six FTC, two medullary thyroid carcinomas and one poorly differentiated thyroid carcinoma (Fig. 1).

The research data included demographic information, ultrasound features, risk stratification and cytologic or surgical pathology for outpatients and inpatients. Repeat FNAB was required as follows: i) if the initial puncture was nondiagnostic or inconclusive cytopathology; ii) if a nodule was suspected of malignancy but with a Bethesda II cytopathology; iii) if a nodule developed new malignant features during the follow-up period; iv) before thermal ablation for patients with an initial Bethesda II nodule.

This study was approved by the Institutional Ethics Committee of Guangdong Provincial People’s Hospital (KY2023-472-01), which waived the requirement for informed consent to review images and medical records.

### Ultrasonography and nodule evaluation

Real-time US examinations were performed using a variety of commercial ultrasound instruments by board-certified radiologists at the Department of Ultrasound. Ultrasound-guided FNAB was subsequently performed by endocrinologists who specialized in the technique. A formal color ultrasound report within 3 months should be provided before a biopsy. A radiologist with more than 20 years of experience in thyroid ultrasound imaging described the ultrasound features of thyroid nodules. Then two experienced endocrinologists recorded the information with the radiologist’s help. All of them were blinded to the FNAB results and final diagnosis. Conclusions were resolved by consensus in case of any disagreement. The ultrasound lexicons of the four TIRADS consist of composition, echogenicity, margin, orientation and calcification. The definitions and classifications of these components are nearly the same for these TIRADS, but there is a little difference (e.g., the definitions of solid, mixed echogenicity and spongiform structure). Nodules were generally classified according to the definitions of each TIRADS during the study.

In our study, nodules were classified as ‘FNAB-indicated’ and ‘non-FNAB indicated’ based on the recommendations of each TIRADS. Missed malignancy biopsy was defined as any case of missed malignant nodule among non-FNAB indicated nodules. Missed diagnosis that may affect prognosis refers to any missed diagnosis of malignant cancer with a biopsy nodule size ≥2.5 cm (5, 12). Unnecessary biopsy was defined as any case of benign nodule among FNAB-indicated nodules in the total number of nodules. Avoided biopsy was defined as any case of benign nodule among non-FNAB indicated nodules in the total number of nodules (13, 14, 15).

### Statistical analysis

Statistical analysis was performed using SPSS 26 software (IBM, USA) and MedCalc 19.0.4 software (MedCalc, Belgium). Continuous data conforming to normal distribution were expressed as means ± SD and range; otherwise, median (interquartile range). Differences between the two groups were evaluated by independent samples *t*-test or non-parametric test. Categorical variables were presented as frequencies (proportions). Differences in the distribution of variables between or among groups were evaluated by chi-squared tests (Pearson chi-square, continuity correction, Fisher’s exact test or McNemar test, as appropriate). Bonferroni correction was applied for post-hoc analysis if multiple comparison tests were encountered. Receiver operation characteristic analysis was used to evaluate the reliability of recommendations for several evaluation methods. Sensitivity, specificity, positive predictive value (PPV), negative predictive value (NPV) and area under the curve (AUC) were calculated for each method. DeLong method was used to compare the AUC of evaluation methods. The statistical significance level was set at *P*-value <0.05. An adjusted significance level of 0.0083 (0.05/6) was also considered for the multiple pairwise comparisons based on the Bonferroni correction test.

## Results

The patients’ mean age was 43.4 ± 12.6 years (range, 12–82). Of 693 patients, 501 (71.7%) were females and 192 (27.5%) were males. Benign nodules were significantly larger than malignant ones (median 2.6 cm (Q1–Q3, 1.8–3.4 cm) vs 1.4 cm (Q1–Q3, 1.1–1.9 cm); *P* < 0.001). The features of composition, echogenicity, margin, orientation and calcification exhibited remarkable disparities between benign and malignant nodules (all *P* < 0.001) (Table 1).

**Table 1:** Demographic characteristics and ultrasound features of the thyroid nodules among patients.

	Total	Benign	Malignant	*P*
Number of nodules	699	448	251	-
Age (years)	43.4 ± 12.6 (12–82)	44.1 ± 12.8 (12–82)	42.3 ± 12.2 (14–80)	0.073
Gender				0.324
Male	192 (27.7)	119 (26.7)	73 (29.6)	
Female	501 (72.3)	327 (73.3)	174 (70.4)	
Size (cm)	2.1 (1.4, 3.0)	2.6 (1.8, 3.4)	1.4 (1.1, 1.9)	<0.001
Ultrasound features				
Composition				<0.001
Solid	526 (75.2)	288 (64.3)	238 (94.8)	
Mixed solid	171 (24.5)	158 (35.3)	13 (5.2)	
Cyst	2 (0.3)	2 (0.4)	0 (0)	
Echogenicity				<0.001
Iso- or hyperechoic	414 (59.2)	386 (86.2)	28 (11.2)	
Hypoechoic	247 (35.3)	59 (13.2)	188 (74.9)	
Marked hypoechoic	38 (5.5)	3 (0.6)	35 (13.9)	
Orientation				<0.001
Taller than wide	54 (7.7)	5 (1.1)	49 (19.5)	
Wider than tall	645 (92.3)	443 (98.9)	202 (80.5)	
Margin				
Smooth	452 (64.7)	407 (90.8)	45 (17.9)	<0.001
Ill-defined	251 (35.9)	53 (11.8)	198 (78.9)	<0.001
Lobulated or irregular	247 (35.3)	41 (9.2)	206 (82.1)	<0.001
Calcification				<0.001
Absent	431 (61.7)	362 (80.8)	69 (27.5)	
Microcalcifications	167 (23.9)	21 (4.7)	146 (58.2)	
Macrocalcifications	90 (12.9)	57 (12.7)	33 (13.1)	
Rim calcification	11 (1.6)	8 (1.8)	3 (1.2)	

Data are presented as numbers (%) or mean ± SD (range).

The malignant rates for the four TIRADSs all demonstrated striking distinctions among categories (all *P* < 0.001). The malignant rates of TIRADS categories were all within the range of the recommendations except ACR-TIRADS TR3 and TR4, C-TIRADS CTR2 to 3 and CTR4b to 5 and EU-TIRADS category 4 (Supplementary Table 1 (see section on Supplementary materials given at the end of the article)). The sensitivity, specificity, NPV, PPV and AUC for the four TIRADS were different using ultrasound-based predictive malignant risk categories. Kwak-TIRADS had the highest sensitivity (93.2%) and NPV (95.7%), while ACR-TIRADS had the highest specificity (96.6%) and PPV (88.8%) (Supplementary Table 2).

### Performance of current TIRADS according to selective power and consistency

Table 2 illustrates the discrepancies in estimated malignant risk and FNAB thresholds for specific risk stratification among TIRADS. The differences in FNAB recommendations were distributed in the low- and intermediate-risk stratification categories. The total unnecessary biopsy rate (UBR) and missed malignancy rate (MMR) based on the indication-based method were found to be significantly different among the four systems (both *P* < 0.001). Pairwise comparison analysis revealed that ACR-TIRADS had the lowest UBR (28.9%), while Kwak-TIRADS demonstrated the lowest MMR (2.6%). Furthermore, ACR-TIRADS exhibited the lowest UBR (53.3%) in the low-risk category, whereas C-TIRADS performed the lowest (40%) in the intermediate-risk category.

**Table 2:** Ability of the four TIRADS to reduce unnecessary biopsies and select eligible nodules for puncture based on FNAB recommendations.

	ACR-TIRADS	Kwak-TIRADS	C-TIRADS	EU-TIRADS
Total UBR	202/699 (28.9%)^a^	296/699 (42.3%)^b,c^	248/699 (35.5%)^a,c^	331/699 (47.4%)^b^
Total MMR	36/282 (12.8%)^a^	4/156 (2.6%)^b^	53/253 (20.9%)^a,c^	46/163 (28.2%)^c^
Benign category	TR1, benign	2, probably benign 0%	CTR2, benign 0%	2, benign
	TR2, not suspicious	3, (0 suspicious US feature)	CTR3, probably benign	
	≤2%	2.0–2.8%	<2%	0%
	No FNAB	No FNAB	No FNAB	No FNAB
UBR	0%	0%	0%	0%
MMR	2/139 (1.4%)	4/156 (2.6%)	1/10 (10%)	0%
Low-risk category	TR3, mildly suspicious	4a, low suspicion for malignancy	CTR4a low suspicion for malignancy	3, low risk
	<5%	3.6–12.7%	2–10%	2–4%
	≥2.5 cm	≥1.0 cm	>1.5 cm	>2.0 cm
UBR	113/212 (53.3%)^a^	222/235 (94.5%)^b^	194/259 (74.9%)^c^	243/348 (69.8%)^c^
MMR	7/94 (7.4%)	0%	15/54 (27.8%)	5/99 (5.1%)
Intermediate-risk category	TR4, moderately suspicious	4b, intermediate suspicion for malignancy	CTR4b, intermediate suspicion for malignancy	4, intermediate risk
	5–20%	6.8–37.8%	10–50%	6–17%
	≥1.5 cm	≥1.0 cm	>1.0 cm	>1.5 cm
UBR	65/133 (48.9%)^a,b^	43/69 (62.3%)^b^	40/100 (40%)^a^	46/78 (59%)^a,b^
MMR	27/49 (55.1%)	0%	10/13 (76.9%)	9/20 (45%)
High-risk category		4c, moderate concern but not classic for malignancy	CTR4c, moderate concern but not classic of malignancy	
		21–91.9%	50–90%	
		>1.0 cm	>1.0 cm	
	TR5, highly suspicious	5, highly suggestive of malignancy	CTR5, highly suggestive for malignancy	5, high risk
	>20%	>95%	>90%	26–87%
	≥1.0 cm	≥1.0 cm	>1.0 cm	>1.0 cm
UBR		27/202 (13.4%) for Kw 4c^a^	13/170 (7.6%) for CTR4c^a^	
	24/215 (11.2%)^a^	4/37 (10.8%) for Kw 5^a^	1/8 (12.5%) for CTR5^a^	42/266 (15.8%)^a^
MMR	0%	0% for Kw 4c and 5	21/23 (91.3%) for CTR4c 1/1 (100%) for CRT 5	32/37 (85.6%)

TIRADS, Thyroid Imaging Reporting and Data System; FNAB, fine-needle aspiration biopsy; values are presented as number (%). UBR, unnecessary biopsy rate; MMR, missed malignancy rate; Kw, Kwak-TIRADS.

The comparison of four systems at total or specific TIRADS category and size levels is based on chi-square tests with post-hoc Bonferroni correction.

a–c represents the results of post-hoc tests at a corrected *P*-value of 0.0083 (0.05/6). The same letters indicate no differences in pairwise comparisons (such as ‘a–a’), while different letters indicate significant differences (such as ‘a–b’).

**Figure 2:**
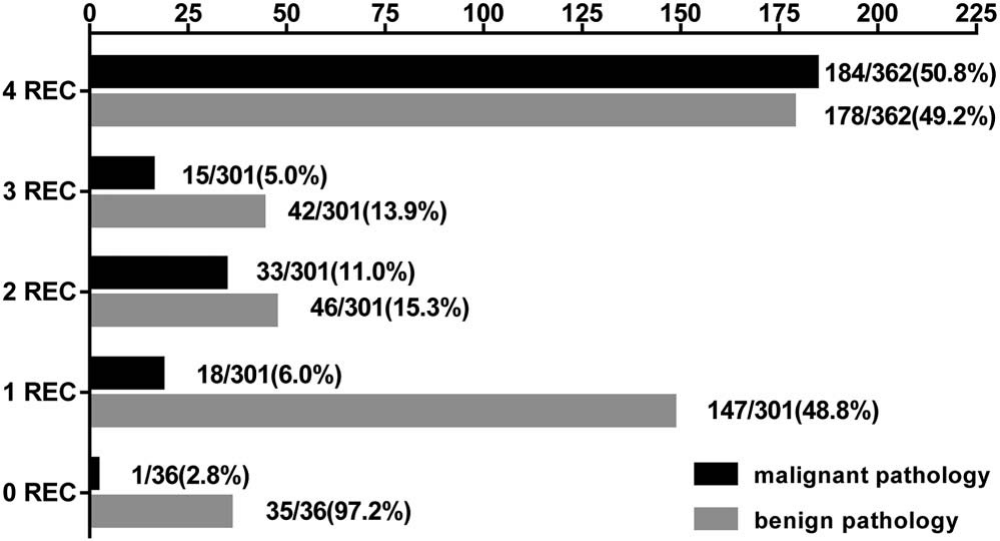
Frequency distribution of malignant and benign nodules in line with FNAB indicated consistency of the four TIRADS. 0–4 REC represents the number of recommendations for FNAB by four TIRADS, from fully recommended (4 REC) to partially recommended (1–3 REC) to not recommended at all (0 REC).

Subsequently, the consistency of TIRADS using an indication-based method was analyzed (Fig. 2). The malignancy rate of FNAB-indicated nodules by all four TIRADS simultaneously was found to be only 50.8%. Inconsistent recommendations were observed for 301 nodules (43.1%). Furthermore, the malignancy rate of the nodules did not exhibit a linear increase from one to three recommendations. Contrary to expectations, the malignancy rate of nodules recommended by three TIRADS (5.0%) was even lower than that of nodules recommended by a single TIRADS (6.0%).

### Impact of using evaluation methods in selection of nodules for biopsy

Table 3 displays the reliability of several evaluation methods in the overall cohort. ACR-TIRADS had the highest baseline AUC (0.703; 95% CI 0.667–0.737), specificity (54.9%; 95% CI 50.2–59.6%) and PPV (51.6%; 95% CI 48.7–54.4%) among the four TIRADS. By adopting serial testing, integrating ACR-TIRADS with Kwak-TIRADS tremendously ameliorated the specificity, PPV and AUC of recommended biopsies. Nevertheless, this approach revealed a decrement in sensitivity and NPV in contrast to Kwak-TIRADS. Notwithstanding the fact that the combination of EU-TIRADS with C-TIRADS suggested conspicuously declining trends in sensitivity (78.5 vs 81.7%, *P* = 0.008), the NPV was immensely elevated in comparison with EU-TIRADS (81.3 vs 71.8%, *P* = 0.019).

**Table 3:** Reliability of biopsy recommendations for new evaluation methods in the overall cohort.

*n* = 699	Sensitivity (%)	Specificity (%)	NPV (%)	PPV (%)	AUC	Note
A	85.7 (80.7–89.7)	54.9 (50.2–59.6)	87.2 (83.3–90.3)	51.6 (48.7–54.4)	0.703 (0.667–0.737)	-
K	98.4 (96.0–99.6)	33.9 (29.6–38.5)	97.4 (93.4–99.0)	45.5 (43.8–47.2)	0.662 (0.625–0.697)	-
C	78.9 (73.3–83.8)	44.6 (40.0–49.4)	79.1 (74.4–83.0)	44.4 (41.8–47.0)	0.618 (0.580–0.654)	-
E	81.7 (76.3–86.3)	26.1 (22.1–30.4)	71.8 (65.2–77.5)	38.2 (36.4–40.2)	0.539 (0.501–0.576)	-
To increase specificity (serial test)* Two-TIRADS						
A–K	85.3 (80.3–89.4)	57.1 (52.4–61.8)	87.4 (83.6–90.4)	52.7 (49.7–55.7)	0.712 (0.677–0.745)	Sig
*P*	1.000	0.002	0.960	0.741	0.0226	Vs. A
	<0.001	<0.001	<0.001	0.028	0.0006	Vs. K
A–C	73.3 (67.4–78.7)	58.9 (54.2–63.5)	79.8 (76.0–83.1)	50.0 (46.7–53.3)	0.661 (0.625–0.696)	NS
A–E	74.5 (68.6–79.8)	57.6 (52.9–62.2)	80.1 (76.3–83.5)	49.6 (46.4–52.8)	0.660 (0.624–0.696)	NS
K–C	78.9 (73.3–83.8)	45.1 (40.4–49.8)	79.2 (74.6–83.2)	44.6 (42.0–47.2)	0.620 (0.583–0.656)	NS
K–E	80.5 (75.0–85.2)	52.0 (47.3–56.7)	82.6 (78.5–86.1)	48.4 (45.6–51.3)	0.662 (0.626–0.697)	NS
E–C	78.5 (72.9–83.4)	52.5 (47.7–57.2)	81.3 (77.2–84.8)	48.0 (45.1–51.0)	0.655 (0.618–0.690)	Sig
*P*	1.000	<0.001	0.509	0.284	<0.0001	Vs. C
	0.008	<0.001	0.019	0.003	<0.0001	Vs. E

Numbers in parentheses are 95% confidence intervals.

A: ACR-TIRADS, K: Kwak-TIRADS, C: C-TIRADS, E: EU-TIRADS, AUC: area under the curve, NS: no significance, Sig.: significance, Vs.: versus; NPV, negative predictive value; PPV, positive predictive value.

* The serial test is defined as follows: the same nodule is recommended for FNAB only when both tests are eligible or not recommended for FNAB when one of the tests is ineligible.

For eligible nodules recommended for biopsies, Kwak-TIRADS combined with ACR-TIRADS could lessen biopsies by approximately 19.2%. In addition, the total UBR considerably lessened from 42.3 to 27.5%, accompanied by the augment in MMR from 2.6 to 12.6%. C-TIRADS combined with EU-TIRADS remarkably lowered the UBR (*P* < 0.05), but the combination of EU-TIRADS with C-TIRADS lessened more biopsies (22.0 vs 7.8%) (Table 4).

**Table 4:** Selective power of new evaluation methods for eligible nodules initially assessed by the four TIRADS.

Methods	Biopsies	Indication	Pathology		Non-biopsies	Benign among biopsies (X)	Missed malignancy among non-biopsies	UBR (X/699)	MMR	Reduced biopsies rate
			Benign	Malignancy						
ACR-TIRADS	417	Indn.	202	215	282	202	36	28.9%^†^	12.8%	-
		Non	0	0						
+Kwak	406	Indn.	192	214	293	192	37	27.5%	12.6%	10/417
		Non	10	1						(2.4%)
Kwak-TIRADS	543	Indn.	296	247	156	296	4	42.3%	2.6%^†^	-
		Non	0	0						
+ACR^#^	406	Indn.	192	214	293	192	37	27.5%^***^	12.6%^***^	104/543
		Non	104	33						(19.2%)
C-TIRADS	446	Indn.	248	198	253	248	53	35.5%	20.9%	-
		Non	0	0						
+EU	410	Indn.	213	197	289	213	54	30.5%^*^	18.7%	35/446
		Non	35	1						(7.8%)
EU-TIRADS	536	Indn.	331	205	163	331	46	47.4%	28.2%	-
		Non	0	0						
+C	410	Indn.	213	197	289	213	54	30.5%^***^	18.7%^*^	118/536
		Non	118	8						(22.0%)

ACR: ACR-TIRADS, Kwak: Kwak-TIRADS, C: C-TIRADS, EU: EU-TIRADS, UBR: unnecessary biopsy rate, MMR: missed malignancy rate, Indn.: indication for fine-needle aspiration biopsy, Non: without indication for fine-needle aspiration biopsy.

^*^ P < 0.05 compared to original TIRADS.

^***^ P < 0.001, compared to original TIRADS.

^#^ The serial test is defined as follows: the same nodule is recommended for FNAB only when both tests are eligible or not recommended for FNAB when one of the tests is ineligible.

^†^ Represents the lowest missed malignancy rates (Kwak-TIRADS, *P* < 0.001) or unnecessary biopsy rates (ACR-TIRADS, *P* < 0.001) among the four original TIRADS in post-hoc pairwise comparisons.

Finally, we evaluated the value of the several methods from the perspective of the impact on the patient’s prognosis (Table 5). In contrast, the preferred methods included combining Kwak-TIRADS and ACR-TIRADS method, combining C-TIRADS and EU-TIRADS method, none of which increased the risks.

**Table 5:** Value of derivational new methods evaluated by missed biopsies that could potentially influence prognosis.

Methods	MMR	Non-biopsies	Missed malignancy among non-biopsies	Missed malignancy ≥2.5 cm*	Missed malignancy ≥3 cm*	Comment
ACR-TIRADS	12.8%	282	36	2	0	
+Kwak	12.6%	293	37	3	1	Not preferred
Kwak-TIRADS	2.6%	156	4	3	1	
+ACR	12.6%	293	37	3	1	Preferred
C-TIRADS	20.9%	253	53	5	3	
+EU	18.7%	289	54	5	3	Preferred
EU-TIRADS	28.2%	163	46	0	0	
+C	18.7%	289	54	5	3	Not preferred

MMR: missed malignancy rate.

* Derived from the white paper of the ACR-TIRADS Committee, they cited SEER analysis data indicating an increased risk of distant metastases with nodules ≥2.5 cm and an increment in 10-year thyroid cancer-specific mortality with nodules ≥3 cm (5, 12).

### Example of new methods interpretation

**Figure 3:**
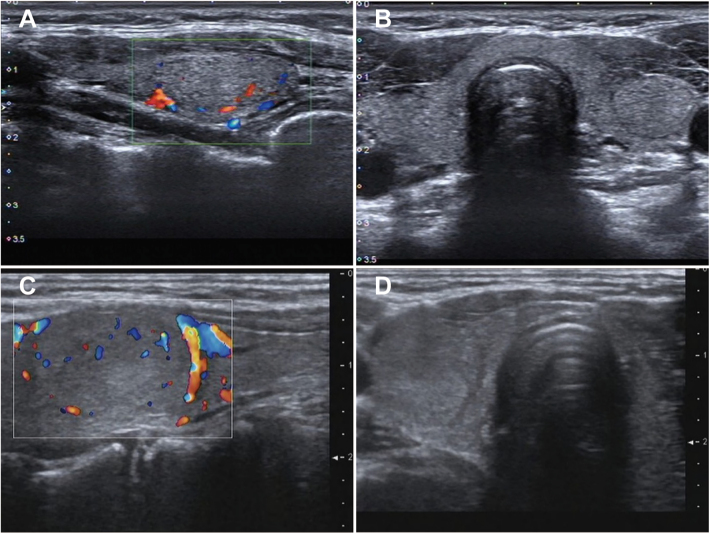
Ultrasound of a 49-year-old female patient with a left thyroid nodule. (A) Longitudinal and (B) transverse images reveal a solid nodule with a maximum diameter of 2.2 cm, regular margins, isoechogenicity and no calcifications features. Kwak-TIRADS had an indication for puncture for category 4a, while ACR-TIRADS did not for category 3 under the 2.5 cm threshold. In accordance with the serial test, FNAB for the nodule is not recommended due to the ineligibility of one of the tests. In practical terms, the patient underwent repeated punctures, both of which were Bethesda II cytopathology. A 29-year-old female patient had a nodule with a maximum diameter of 1.7 cm. (C) Longitudinal and (D) show features as follows: solid composition, isoechogenicity and regular shape without calcifications. The nodule is classified as 4b by C-TIRADS, which had indications, while it was not recommended by EU-TIRADS with category 3, which is below the puncture threshold of 2.0 cm. Likewise, the nodule could not be recommended for FNAB based on the serial test. The cytopathology was finally proven to be Bethesda II category.

Figure 3 depicts two examples of combining two TIRADS to optimize puncture recommendations. The first case is a solid nodule with no other malignant features. It is recommended for biopsy by the Kwak-TIRADS as a 4a category with the diameter of the nodule exceeding 1 cm. Nevertheless, it is not recommended by the ACR-TIRADS that the total score is 3 points, the risk level was categorized as TR3, and the diameter of the nodule does not exceed 2.5 cm. The features of the second nodule are similar to the first case. The C-TIRADS category is 4a. The maximum diameter of the nodule exceeds 1.5 cm, so there is an indication for puncture. Nonetheless, it was categorized as 3 by the EU-TIRADS, which is ineligible owing to the fact that the maximum diameter does not exceed 2 cm. In both cases, there is no indication for puncture through serial testing, which aligns with the benign cytopathology results. Both the validation and performance of the combined method of the two TIRADS are supported by these results.

## Discussion

Our study demonstrated significant differences among the four TIRADS recommended for biopsy across two dimensions: selective power and consistency analysis. Subsequently, we constructed several two-TIRADS combined methods and demonstrated their good performance. Finally, the value of the new methods was validated by introducing the frequency of missed diagnoses that may affect prognosis.

First, as evidenced by previous studies, a desirable strategy for improving the performance of TIRADS was to maintain high accuracy and specificity, lessen the unnecessary biopsy rate, and not immensely lower sensitivity (16). Our study holds a pertinent conclusion that even with some sensitivity reduction, the missed biopsy would still be better controlled without a significant decrease in NPV (e.g., the EU-TIRADS and C-TIRADS combined method), which is primarily attributable to the fact that NPV has a complementary association with the missed malignancy rate. Apart from that, our exploration findings have been validated in the Clotilde study, thereby suggesting NPV as a crucial characteristic indicator for TIRADS evaluation (17).

The accuracy of various TIRADS recommended for biopsy is hardly comparable to their excellent ability to distinguish benign from malignant nodules. In accordance with other studies, our study found the lowest missed malignancy rate for Kwak-TIRADS, which may be attributed to its good sensitivity, NPV and lower size threshold (18). The commonly used ACR-TIRADS performed the best in terms of the UBR, similar to many studies (19, 20). In addition, almost all nodules that could be altered for recommendations are primarily located in low- and moderate-suspicion categories across the four TIRADS due to more consistent recommendations for high-suspicion categories. The consistency analysis for FNAB also showed an unsatisfactory accuracy (approximately 50%) for multiple TIRADS, making a unanimous recommendation.

Differences in population characteristics, lexicon and risk stratification may partly explain these discrepancies (21). From a reductionist perspective, the main factors involve the two most fundamental elements of nodular features and size, which are interactive rather than independent when recommending a biopsy. Each feature has a different weight and significance in the TIRADS. However, the nodular FNAB threshold is more changeable than the risk stratification corresponding to specific nodule features, which have a greater impact on the TIRADS’ performance (22). In fact, methods to alter FNAB thresholds are common (18, 23). To our knowledge, only ACR-TIRADS has disclosed its reason for FNAB thresholds, and the uncertainty of the cut-off value may also contribute to heterogeneity (5).

Second, the performance of the two-TIRADS combined method in serial was found to significantly reduce the UBR, which essentially improves the specificity through serial testing. Regardless of the combination order, multiple combinations significantly reduced the UBR compared to the original TIRADS. For instance, the combination of EU-TIRADS and C-TIRADS substantially heightened the UBR and lessened biopsies by 22.0%, which was probably ascribable to a substantial proportion of eligible low-risk nodules in EU-TIRADS being distributed in C-TIRADS category 3 (approximately 40%), thereby demonstrating no recommendation value for biopsy. The integration of Kwak-TIRADS with ACR-TIRADS has yielded remarkable benefits, which was likely attributable to the notable discrepancy in size thresholds between the two systems - 2.5 cm for ACR-TIRADS and 1.0 cm for Kwak-TIRADS - in the category of low-suspicion nodules. It is particularly noteworthy that this disparity has brought about a reduction of up to 37% in biopsy rates for nodules that meet the criteria defined by Kwak-TIRADS. These results were corroborated by other studies, demonstrating that the closer the biopsy size criteria, the more similar the unnecessary biopsy rates, and vice versa (24).

The new methods offer the advantage of not requiring additional indicators or tools, facilitating their application in resource-limited settings. Moreover, the different habits of using various types of TIRADS in different regions are taken into consideration (25). To increase efficiency, the utilization of worksheets or apps to aid assessment may be considered.

Third, our study introduced a novel framework for evaluating the reliability of methods that improve the UBR, encompassing both quantitative and qualitative indicators of missed diagnosis. This is unsurprising, as setting FNAB thresholds was partly motivated by the desire to avoid missed diagnoses that affect prognosis. Furthermore, the UBR and MMR exhibit a reciprocal relationship, and an effective strategy for improving performance necessitates an appropriate balance between them (26). Nevertheless, the issue of missed diagnoses is often overlooked.

The present study is the first to adopt more stringent criteria for missed diagnoses, which would eliminate methods that may affect prognosis. The assumption was made that no missed cases with additional impact on prognosis could be tolerated (i.e., ‘no harm’), which warrants further discussion in the future (). Moreover, although the ACR-TIRADS’ cut-off value of ≥2.5 cm potentially affects cancer prognosis, as confirmed in a subsequent large-scale cohort, validation in other cohorts is still required (10). Nevertheless, with the current criteria cited by the ACR-TIRADS Committee, suitable methods could still be identified from this study (5). The directionality of the two-TIRADS combined method must be considered, such as the combination of Kwak-TIRADS with ACR-TIRADS rather than the reverse.

Our study has some limitations. First, this was a retrospective, single-center study and the study’s results need to be validated in a prospective multicenter cohort study. Second, we applied strict criteria considering repeated FNAB as benign pathology, which inevitably introduced selection bias and limited the sample size. However, it is challenging to avoid about 1–2% of false-negative results that prove to be malignant (27). Third, the malignancy rate of samples may affect the performance of diagnostic tests, so the conclusions of this study still need to be verified in samples with different malignancy rates and the distribution of nodules in the future. Moreover, the decision to perform FNAB should also consider the patient’s risk factors for thyroid cancer, anxiety status, comorbidities and other relevant factors. In addition, nodules <1 cm were not included in this study, and their management usually requires the evaluation of abnormal cervical lymph nodes or high-risk malignant features.

## Conclusion

The present study identified opportunities for improving the UBR of inconsistently recommended nodules among multiple TIRADS. Several two-TIRADS combined methods proved to be effective and safe for achieving this goal within the existing TIRADS framework. Furthermore, this study emphasizes the necessity of evaluating the quality of missed diagnoses when attempting to reduce unnecessary biopsies. Given the current situation, the establishment of a uniform international guideline for FNAB puncture recommendations is called for.

## Supplementary materials



## Declaration of interest

The authors declare that the research was conducted in the absence of any commercial or financial relationships that could be construed as a potential conflict of interest.

## Funding

This work was supported by the Science and Technology Plan of Guangzhou under Grant No. 202103000048 and 202201011500.
