# Supplementary material for: Optimizing the screening process for TIRADS could reduce the number of unnecessary thyroid biopsies
Source: Endocr Connect. 2025 Mar 26;14(5):e250097. doi: 10.1530/EC-25-0097 (PMC11964483; doi:10.1530/EC-25-0097)
Supplement: Supplementary file 1 [file supplementary_materials.pdf]

Supplementary Table 1 Comparison of estimated malignant risks with several TIRADS

		Final diagnosis		Recommended	Estimated	*P*
	Total	Benign	Malignancy	malignant risk	malignant risk	
	n=699	n=448	n=251	(%)	(%)	
ACR-TIRADS						<0.001
TR2	139(19.9)	137(30.6)	2(0.8)	≤2	1.4	
TR3	212(30.3)	200(44.6)	12(4.8)	<5	5.7	
TR4	133(19.0)	87(19.4)	46(18.3)	5-20	34.6	
TR5	215(30.8)	24(5.4)	191(76.1)	> 20	88.8	
Kwak-TIRADS						<0.001
3	156(22.3)	152(33.9)	4(1.6)	2.0-2.8	2.6	
4a	235(33.6)	222(49.6)	13(5.2)	3.6-12.7	5.5	
4b	69(9.9)	43(9.6)	26(10.4)	6.8-37.8	37.7	
4c	202(28.9)	27(6.0)	175(69.7)	21-91.9	86.6	
5	37(5.3)	4(0.9)	33(13.1)	88.7-97.9	89.2	
C-TIRADS						<0.001
CTR2	10(1.4)	9(2.0)	1(0.4)	0	10	
CTR3	152(21.7)	147(32.8)	5(2.0)	≤2	3.3	
CTR4a	259(37.1)	233(52.0)	26(10.4)	2-10	10.0	
CTR4b	100(14.3)	43(9.6)	57(22.7)	10-50	57.0	
CTR4c	170(24.3)	15(3.3)	155(61.7)	50-90	91.2	
CTR5	8(1.2)	1(0.3)	7(2.8)	≥90	87.5	

EU-TIRADS						<0.001
2	7(1.0)	7(1.6)	0(0)	0	0	
3	348(49.8)	337(75.2)	11(4.4)	2-4	3.2	
4	78(11.2)	57(12.7)	21(8.4)	6-17	26.9	
5	266(38.0)	47(10.5)	219(87.2)	26-87	82.3	

Note. Data are presented as numbers (%)

Supplementary Table 2 Diagnostic performance of the four TIRADS according to US-based predictive malignant risk categories

	Sensitivity (%)	Specificity (%)	PPV (%)	NPV (%)	AUC
ACR-TIRADS	76.1	96.6	88.8	87.6	0.923
	(70.3-81.2)	(92.1-96.5)	(84.3-92.2)	(85.0-89.8)	(0.900-0.941)
Kawk-TIRADS	93.2	83.5	76.0	95.7	0.925
	(89.4-96.0)	(79.7-86.8)	(71.9-79.6)	(93.3-97.2)	(0.903-0.944)
C-TIRADS	87.3	86.8	78.8	92.4	0.908
	(82.5-91.1)	(83.3-89.8)	(74.4-82.5)	(89.8-94.4)	(0.885-0.929)
EU-TIRADS	87.3	89.5	82.3	92.6	0.913
	(82.5-91.1)	(86.3-92.2)	(78.0-86.0)	(90.1-94.5)	(0.890-0.933)

Note. Numbers in parentheses are 95% confidence intervals. PPV: Positive predictive value, NPV: Negative predictive value, AUC: Area under the curve
